# Workflow towards automated segmentation of agglomerated, non-spherical particles from electron microscopy images using artificial neural networks

**DOI:** 10.1038/s41598-021-84287-6

**Published:** 2021-03-02

**Authors:** Bastian Rühle, Julian Frederic Krumrey, Vasile-Dan Hodoroaba

**Affiliations:** 1 Federal Institute for Materials Research and Testing (BAM), Richard-Willstätter-Strasse 11, 12489 Berlin, Germany; 2 Federal Institute for Materials Research and Testing (BAM), Unter den Eichen 44-46, 12203 Berlin, Germany; 3 Faculty IV - Electrical Engineering and Computer Science, Technical University of Berlin, Marchstrasse 23, 10587 Berlin, Germany

**Keywords:** Imaging studies, Nanoparticles, Nanoparticles

## Abstract

We present a workflow for obtaining fully trained artificial neural networks that can perform automatic particle segmentations of agglomerated, non-spherical nanoparticles from scanning electron microscopy images “from scratch”, without the need for large training data sets of manually annotated images. The whole process only requires about 15 min of hands-on time by a user and can typically be finished within less than 12 h when training on a single graphics card (GPU). After training, SEM image analysis can be carried out by the artificial neural network within seconds. This is achieved by using unsupervised learning for most of the training dataset generation, making heavy use of generative adversarial networks and especially unpaired image-to-image translation via cycle-consistent adversarial networks. We compare the segmentation masks obtained with our suggested workflow qualitatively and quantitatively to state-of-the-art methods using various metrics. Finally, we used the segmentation masks for automatically extracting particle size distributions from the SEM images of TiO_2_ particles, which were in excellent agreement with particle size distributions obtained manually but could be obtained in a fraction of the time.

## Introduction

One of the most fundamental—and indeed one of the defining—properties of a nanomaterial is the size of its constituent components. Typically, any physical–chemical characterization of such a material starts with the accurate measurement of the size and size distribution and continues with the determination of other relevant parameters such as shape, structure, crystallinity, chemical composition, specific surface area, and surface chemistry^1^. However, even a deceptively simple measurement such as a particle size can constitute a challenging analytical task, especially when the sample is polydisperse and the nanoparticle morphology deviates from an ideal, spherical shape. The problem of accurate size measurements is further aggravated by the fact that different sizing techniques often measure different quantities. For example, ensemble sizing methods such as dynamic light scattering (DLS), particle tracking analysis (PTA), or small-angle X-ray scattering (SAXS) typically measure a hydrodynamic size from a volume- or intensity-weighted size distribution, however, according to the Recommendation on the Definition of Nanomaterials by the European Commission, the number-based particle size distribution should be considered when classifying a material as a nanomaterial^2^.

On the other hand, electron microscopy techniques yield images from which the shape and size of individual nanoparticles (or at least their 2D projections) can be extracted directly to yield a number-based particle size distribution. This advantage is however counterbalanced by the reduced number of particles that can be imaged simultaneously, and especially the tedious and usually manually performed extraction of the size and shape descriptors of the nanomaterials from these images. To mitigate these drawbacks, it is highly desirable to use automated image analysis, rather than a tedious manual measurement of individual particles that is prone to errors and operator bias in order to get representative particle size and shape descriptors for a statistically relevant number of nanoparticles.

For “ideal” nanoparticles with simple morphology, i.e. (nearly) spherical, monodisperse particles in a non-agglomerated state such as silica or gold nanoparticles, the automated measurement of the size distribution from electron microscopy micrographs works accurately^3,4^. It has been shown that the degree of nanoparticle agglomeration or aggregation can alter the final result of the particle size distribution considerably^5^, yet a sample preparation procedure yielding isolated, contamination-free nanoparticles that are dispersed homogeneously on a suitable substrate typically requires well-developed protocols which are often not available or work only partially for new or industrial nanoparticles^5,6^.

In recent years, artificial neural networks (ANNs) and especially convolutional neural networks (CNNs) have shown enormous potential in complex computer vision tasks such as image classification and segmentation, and their scope has been extended towards automated image analysis of biomedical and life science data including light microscopy images, cryo TEM, or CT/MRI tomography^7–13^. The drawback of such deep learning algorithms is the need for large training (and ideally also validation) datasets containing high quality annotated images to train the algorithms via backpropagation in supervised learning. In the case of scientific images, such annotated data typically has to be generated manually by trained personnel, which is a very tedious and time-consuming task that binds important resources. Recently, methods that can help predicting labels of an unlabeled target domain were proposed, however, they usually still require a large annotated source domain^14,15^. While there are annotated datasets available for different life science applications such as cell segmentation or neuronal connectomics^16–18^, there is currently still a severe lack of high-quality annotated image data for training, validating, and comparing different algorithms for the segmentation of nanoparticles in scanning electron microscopy (SEM) images, despite the ubiquitousness of electron microscopy in the field.

In some favorable cases, the necessary ground truth segmentation masks for nanoparticles can be generated automatically from SEM in transmission mode (STEM-in-SEM, or simply TSEM) images that are taken from the same sample areas as the SEM images that are used as input during training and inference. Generally, automatic thresholding which is necessary for generating binary segmentation masks is easier with TSEM images than with SEM images due to the difference in contrast generation in both image types. In TSEM, mainly primary electrons transmitted through the material are used for signal generation, and particles generally appear dark on a bright background with particles of similar thickness giving similar contrast (provided they are comprised of the same material)^19^. In SEM on the other hand, secondary electrons (coming rather from the sample surface) are used for signal generation, leading to a more complex contrast formation, strongly dependent on surface morphology, and hence the signal intensity can vary significantly between similar particles within one sample or even within a single particle due to e.g., charge buildup, shadowing, or edge effects, often making thresholding with just a single, automatically calculated threshold value impossible. In more complex cases, a (semi-) automated segmentation of the corresponding TSEM images might however fail as well. This is often the case when several particles are touching or lying on top of each other, a problem that is regularly faced when imaging non-ideal samples. The nonapplicability of fully automated segmentation for non-ideal nanoobjects such as cellulose nanocrystals holds also true for evaluation in TEM^20^. In all these cases, the automated generation or simulation of realistic-looking SEM images together with their ground truth segmentation masks could help tremendously with providing the necessary large amounts of training data that are not readily available by any other means.

We examined the use of generative adversarial networks (GANs) for the generation of training data, i.e., images and their corresponding ground truth segmentation masks, and present a workflow that can give a fully trained CNN for SEM image segmentation with minimal user interaction due to the unsupervised learning of the GANs (typically the process requires less than 15 min hands-on time by the user, with the entire process taking less than 12 h on a single GPU even without optimizing the code and hyperparameters for performance). We compare the segmentation results of the so trained neural network with those of networks trained on automatically generated masks from TSEM images and manually annotated masks using different metrics. Furthermore, we suggest the use of a simple classifier network to obtain particle size distributions from the segmentation results without the need for manual post-processing or filtering of the segmentation masks. Lastly, the provided high-quality SEM images together with their manually annotated ground truth segmentation masks can serve as training or validation data in the future and are a valuable resource for further developing and comparing novel nanoparticle segmentation methods from SEM images.

## Results and discussion

**Figure 1:**
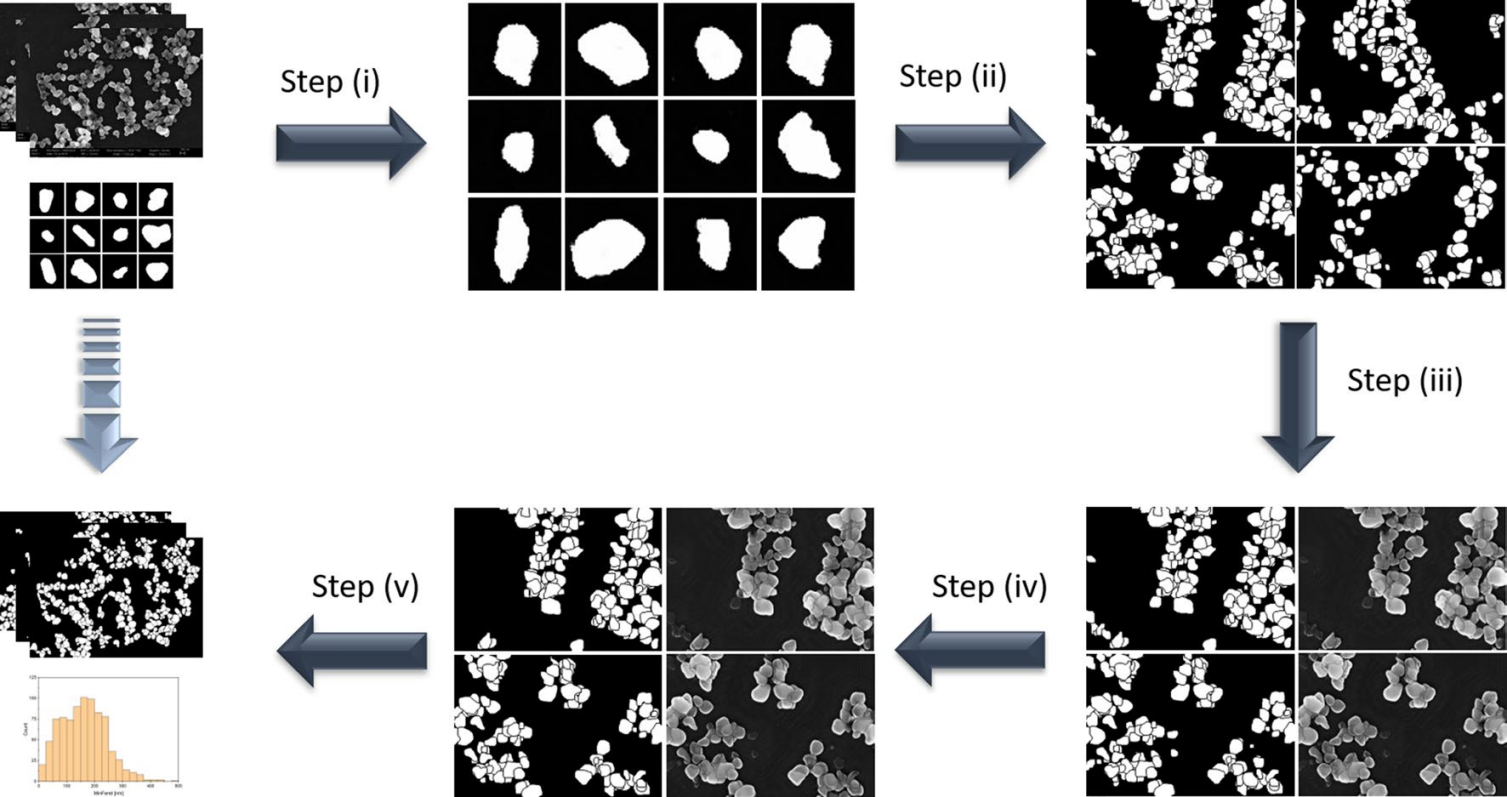
Simplified schematic illustration of the workflow. Data in the top left panel, i.e., scanning electron microscopy micrographs and some representative outlines of particle shapes have to be provided by the user, all other steps are done automatically. In step (i) a Wasserstein GAN is used to produce more particle shapes similar to those provided by the user. In step (ii), they are assembled into fake segmentation masks. In step (iii), a cycleGAN is trained on real SEM images and the fake masks generated in the previous step. In step (iv), artifacts are removed, yielding a set of realistic-looking, fake SEM images and their corresponding segmentation masks. In step (v), a MultiRes UNet segmentation model is trained on these fake data and finally used to produce segmentation masks that can be used to extract further information such as particle size distributions from the real SEM images.

The workflow we investigated for obtaining fully trained neural networks for SEM image segmentation is depicted schematically in Fig. 1. It consists of five main steps: (i) generating random instances of segmentation masks of individual, isolated particles with the desired morphology using a Wasserstein GAN (WGAN)^21,22^, (ii) assembling several of these individual particle masks into masks containing overlapping and agglomerated particles, as typically found in SEM images of real-world samples, (iii) using unpaired image-to-image translation of a cycleGAN to transform these masks into realistic-looking, fake SEM images showing particles that correspond to the instances from the previously generated segmentation masks^23^, (iv) filtering of these images to remove “artifact” particles, i.e., instances in which a feature in the segmentation mask does not correspond to a useful feature in the translated image, (v) using the so obtained data for training a MultiRes UNet for the actual image segmentation task^7,24^.

### UNets for image segmentation

We decided to use the well-established UNet architecture for the segmentation network in the last step, since it has previously been demonstrated that these networks perform very well in biomedical image segmentation^7,24^, however, any image segmentation network can be used here. In a classic UNet, the input image is downsampled by passing it through an encoder consisting of several blocks of convolutional and max pooling layers to obtain the corresponding feature vectors in the “bottle neck”. These are then upsampled again to the original input size by a decoder that consists of blocks of transposed or strided convolutional layers combined with regular convolutions. Skip connections between the encoder and decoder path of the network help to preserve higher level spatial features which can otherwise be lost during the pooling operations. These skip connections are realized by adding or concatenating the output of each block in the encoder to the output of the corresponding block in the decoder at different depths. The additional spatial feature maps are then further up-propagated through successive convolutional layers and blocks in the decoder^6^. The MultiRes UNet architecture improved upon the classical UNet by replacing the regular blocks consisting of two 3 × 3 convolutions by so-called MultiRes blocks that were inspired by Inception blocks^25^ to reconcile features from different context size. However, instead of performing 3 × 3, 5 × 5, and 7 × 7 convolutions in parallel and concatenating the results, the more expensive convolutions with the larger filters were replaced by successive 3 × 3 convolutions, and a residual connection with a 1 × 1 convolution was also added. Additionally, the direct skip connections in regular UNets were replaced with Res Paths, in which the encoder features are passed through a sequence of additional convolutional layers featuring residual connections to help reduce the semantic gap between encoder and decoder^12^.

### Generative adversarial networks for image generation

For the supervised training of such a segmentation network, a set of paired images is required, i.e., an SEM image and its corresponding ground truth segmentation mask, in which each individual particle in the image is outlined. To avoid the tedious task of manually outlining tens or even hundreds of particles in dozens of SEM images, we decided to use GANs for the automated generation of the training data. In a typical GAN architecture, a generator network and a discriminator network are trained against each other in an unsupervised learning process. In simplified terms, given a set of “real” images, the generator network tries to imitate these and generate realistic looking, fake images, while the discriminator network tries to identify which images are real and which ones are fake. Hence, all this approach requires is a set of representative real data for training the GAN. In the case of SEM images, these “real” data are simply a representative set of SEM images of the sample to be analyzed that can be used “as is”, without the need for any further processing or user interaction besides the acquisition of the images. Using these as input, a “regular” GAN could be trained to generate real-looking, fake SEM images. However, since regular GANs typically use random noise rather than meaningful data as input for generating the fake images, the so obtained images could not be used for training the segmentation network directly because of the missing ground truth segmentation masks. Hence, while this approach can be interesting for augmenting the training data set, it does not eliminate the need for manually annotating the data to generate the corresponding segmentation masks. Similarly, training a second GAN to generate fake segmentation masks would not be useful in this case either, because the randomly generated segmentation masks would not necessarily correspond to the randomly generated SEM images of the first GAN, even if the same noise data were used as input.

The key to solving this problem and obtaining automatically generated, fake SEM images together with their corresponding segmentation masks lies in using unpaired image-to-image translation, e.g., via a cycle-consistent generative adversarial network (cylceGAN)^23^. “Unpaired” means here that the individual particle instances in the segmentation masks do not have to correspond to the actual particles in the SEM images, i.e., all that is required for training are representative SEM images of the sample and exemplary segmentation masks. This makes it possible to use randomly generated segmentation masks, as long as they are sufficiently realistic looking, i.e., sufficiently similar to what an actual segmentation mask of the SEM images would look like. Since a segmentation mask is essentially just a 2-bit image of randomly distributed particle outlines, it is usually straightforward to obtain realistic looking segmentation masks through very basic computer simulations. In simple cases where the particles have a well-defined morphology, such masks can readily be generated by randomly distributing white polygons over a black background, possibly with slight variations in size and some random noise added to the vertices to account for small deviations from the perfect shape that are typically found in realistic samples. For more complex particle shapes that are hard to describe by “simple” polygons, it can be more convenient to use a regular GAN to produce the simulated segmentation masks. Since cycleGAN uses unpaired image-to-image translation, the masks can be generated from random noise as no correlation or correspondence of the masks and the actual SEM images are necessary. While in principle a GAN could be trained to generate such masks directly from random noise, its training would require real segmentation masks which in turn have to be obtained manually by annotating SEM images, a process we want to avoid. Hence, it is more convenient to use a GAN for generating masks of single particles which are then assembled in a second step into more complex segmentation masks with several overlapping or agglomerated particles to resemble real-world data, which are then passed on to the cycleGAN for training.

### Generating fake segmentation masks with a WGAN

In this first step, which is also the only step of the workflow requiring user interaction, some randomly chosen particles with representative shapes need to be outlined by the user for training the first GAN. However, by no means does this outlining process of the particles have to be perfect or pixel-precise, it is sufficient to produce an outline that does not deviate too much from what a “typical” particle looks like. Also, it is not necessary to outline hundreds of particles in dozens of images, in our test case we used only 40 representative particle masks for training the GAN, which can be obtained in less than 15 min even by untrained personnel. Augmenting these 40 images by including horizontally and/or vertically flipped versions during training was sufficient to train the GAN to produce a large variety of differently shaped particle masks, as demonstrated in Fig. S2a (Supporting Information). We used a Keras implementation^26^ of a WGAN^21^ with gradient penalty^22^ for generating the individual particle segmentation masks, but other architectures or algorithms will most likely work in this step as well. Next, a random number of such particle masks was generated by the Wasserstein GAN (WGAN) that roughly corresponds to a typical number of particles in the corresponding SEM images (see Fig. S2a in the Supporting Information). We tried to keep this step rather general by using a relatively broad distribution of particles per image (100 to 150) sampled from a uniform distribution, which is just a rough estimate from a quick visual inspection of the input images rather than the result of careful particle counting. It is however better to overestimate rather than underestimate the number of particles per image in this step, because “artifact” particles in the segmentation masks are much easier to remove automatically than artifacts in the simulated SEM images, as will be explained in the next section. We then applied an additional scaling factor sampled from a normal distribution to each generated particle mask. In principle, this additional scaling factor is not strictly necessary. The WGAN already produces differently sized and shaped particle masks as long as “representative” particle outlines were provided by the user for training, i.e., outlines of differently shaped and sized particles. However, since the user provides only a small number of particle masks (e.g., 40), we decided to broaden this distribution further with this additional scaling factor to account for a larger variety of particle sizes. Again, to keep the approach general, we sampled the scaling factors from a broad normal distribution centered around 1.0 with a standard deviation of 0.1 and a cut-off at 3 sigma, but if *a priory* information about the particle size distribution is available to the user, the distribution function can be adjusted accordingly (e.g., using a log-normal distribution or changing the standard deviation according to sample polydispersity). Finally, the rescaled individual particle masks were assembled into an image of appropriate size. To simulate particle agglomeration and aggregation which is normally observed in real-world samples, we used 2D Perlin noise^27^ for distributing the particles over the image. Some examples of simulated real and fake segmentation masks are provided in Fig. S2b (Supporting Information). It should be noted that the exact choice of the parameters did not have a great influence on the final outcome in our case. In an earlier version of the algorithm, we simply used a broad uniform distribution for the additional scaling factors and distributed the particles completely randomly over the whole mask. While this resulted in somewhat less realistic-looking simulated segmentation masks and images due to less pronounced agglomeration and aggregation and also more “artifact particles” in the images, the qualitative and quantitative results after automatically removing these artifacts were very similar.

### Generating fake SEM images with a cycleGAN

In the next step, the segmentation masks obtained as described above were used together with representative, unprocessed SEM images of the sample to train the cycleGAN. After training, the cycleGAN network is in principle able to “translate” images from one domain to another and back. This means, given a randomly generated, fake segmentation mask as input, it can generate fake SEM images that feature particles corresponding to the instances in the segmentation masks, and conversely, it can also generate segmentation masks from real SEM images.

**Figure 2:**
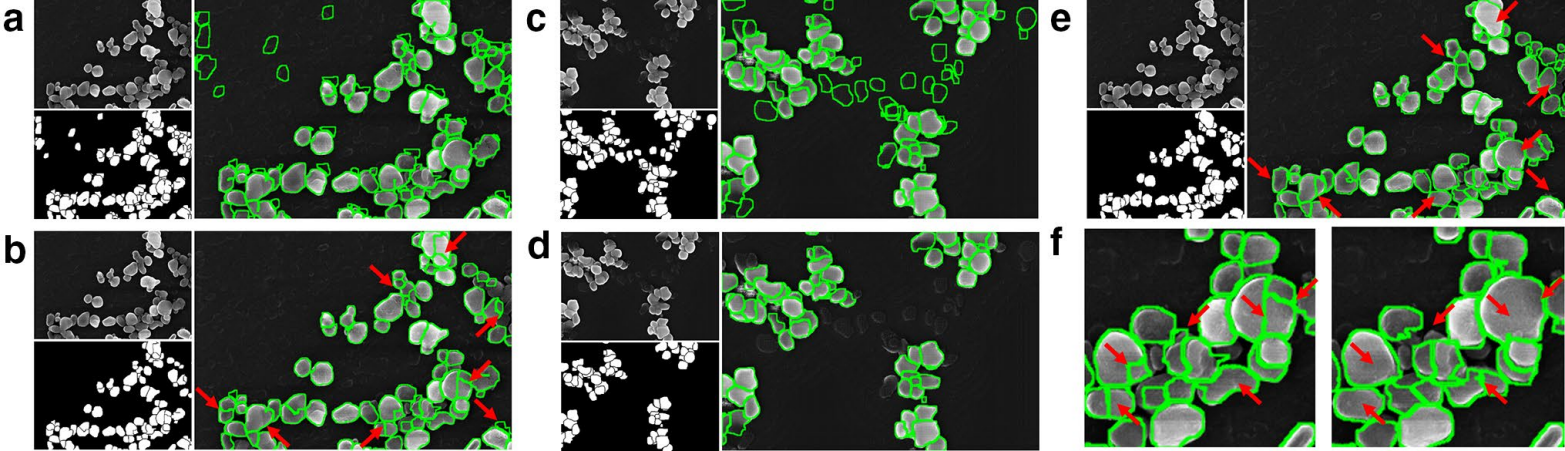
(**a**) Part of a real SEM image, the segmentation mask produced by cycleGAN, and the overlay. The image contains “artifact” particles, i.e., wrongly identified particle instances that only contain background pixels. (**b**) Same images as in (**a**) after a filtering step to remove artifacts. The superfluous particles were successfully filtered out, but there are still some regions with questionable segmentations, highlighted by the red arrows (see also left panel in subfigure **f**). (**c**) Example of a fake SEM image generated by cycleGAN from the corresponding fake segmentation mask. As can be clearly seen in the overlay, the mask contains particle instances that are not present in the simulated SEM image. (**d**) The same image as in (**c**) after filtering. Since both the masks and the corresponding SEM images are computer generated, a virtually unlimited amount of such training data can be generated automatically for use in supervised learning of a segmentation network. (**e**) Segmentation results of the same real SEM image as in (**a**), produced by a UNet segmentation network after training on simulated data. The segmentation in the problematic areas looks more feasible. f) Zoom-in showing some of the problematic segmentations from cycleGAN (left) and the results from MultiRes UNet (right) for comparison. See also Tables 1 and S1 for quantitative comparisons.

The latter is actually the final goal of the workflow, and the segmentation masks that were generated by the cycleGAN from real SEM images indeed identified some particles correctly. However, they also contained a lot of “artifact” particles where the network identified particles in the image that contained in fact only background pixels (see Fig. 2a). The same problem could also be observed when using the GAN for the inverse process of generating fake SEM images from simulated segmentation masks. Nevertheless, in the current case, these artifacts could easily be filtered out based on their mean intensity values. Given the difference in contrast between foreground and background, we simply removed the masks that correlate with dark areas of the image. For filtering, we used the Li threshold value^28^ and compared it to the mean brightness value of each particle instance, but in other cases other methods such as Otsu thresholding^29^ or simply the mean or median values could be used for automatically calculating appropriate threshold values based on the brightness histograms. After this filtering step, the segmentation masks seemed generally much more feasible, but upon closer inspection, we still found that they contained wrong or unprecise segmentations which are not caused by artifact particles, but rather by the segmentation itself (see Fig. 2b,f, and Tables 1 and S1 for qualitative and quantitative comparisons, respectively).

**Table 1:** Comparison of the models discussed in the text according to different metrics.

Model	Training data	Avg IoU (Img)^a^	Avg IoU (Inst)^b^	Avg Youdens Index^c^	Pixel error^d^	Warp Error^e^	FG-rest. Rand error^f^	FG-rest. rand error a. t.^g^	FG-rest. IT score error a. t.^h^
GAN	Simulated	0.7373	0.6111	0.7681	0.9098	0.0014	0.9526	0.8476	0.3298
UNet	Simulated	0.8108	0.6544	0.8546	0.7979	**0.0010**	0.9556	0.8474	0.3379
UNet	TSEM	0.8782	0.6714	0.9286	0.0537	0.0081	0.8638	0.7169	0.2059
UNet	Manual	**0.9070**	**0.7947**	**0.9473**	**0.0418**	0.0039	**0.1986**	**0.1986**	**0.0512**

^a^Average IoU score calculated for the entire image. Value between 0 and 1, higher is better.

^b^Average IoU score calculated separately for each particle instance. Value between 0 and 1, higher is better.

^c^Average Youden’s Index. Value between 0 and 1, higher is better.

^d^Minimum pixel error. Value between 0 and 1, lower is better.

^e^Minimum splits and mergers warping error. Value between 0 and 1, lower is better.

^f^Minimum foreground-restricted Rand error. Value between 0 and 1, lower is better.

^g^Minimum foreground-restricted Rand error after border thinning. Value between 0 and 1, lower is better.

^h^Minimum foreground-restricted information theoretic score error after border thinning. Value between 0 and 1, lower is better.

Given the comparatively simple architecture of the generator in the cycleGAN, we assumed that a dedicated segmentation network with a more complex architecture such as a MultiRes UNet would likely give better results in the final step. Thus, we assessed the possibility of using the cycleGAN to produce a large amount of training data, i.e., realistic looking, fake SEM images from a (virtually unlimited) pool of randomly generated segmentation masks. The generated SEM images look very convincing (see Fig. 2c), and several of the particles in those images correspond well with the instances in the segmentation mask. However, we again also observed several instances of “artifact” particles and for this reason, the generated images could not be used directly for training the segmentation network. Likely, those artifacts are the result of particle instances in the randomly generated masks that are “untypical” in their size or agglomeration state and are hence suppressed by the GAN in the simulated SEM images. While it is possible to minimize their occurrence by carefully fine-tuning the parameters such as the number of particles in each image and the parameters for the Perlin noise during mask generation, this process can be rather tedious and require more user interaction, so we opted again for the automatic filtering procedure explained above for removing the artifacts. This resulted in a virtually unlimited amount of ground truth (GT) segmentation masks and corresponding fake SEM images that can be used to train the final segmentation network (see Fig. 2d). We chose a MultiRes UNet for the final step, however, any image segmentation network architecture that relies on supervised learning can be used here; once the training dataset is obtained following the previous steps, it is very easy to experiment with and optimize the final segmentation network. For comparison with the cycleGAN segmentation in Fig. 2a, the segmentation result produced by a UNet segmentation network after training on 1000 simulated images is shown in Fig. 2e and f.

### Quantitative assessment of the segmentation results

To assess the performance of the fully trained networks more quantitatively, we evaluated their performance based on different metrics for six images from the validation dataset. The assessed models were: the generator model of the cycle GAN, a MultiRes UNet model trained on automatically generated images and segmentation masks obtained by the process outlined above, a MultiRes UNet model trained on segmentation masks that were generated from corresponding TSEM images (see Supporting Information for details), and finally a MultiRes UNet model trained on manually annotated segmentation masks as the gold standard. The same hyperparameters, image augmentations, loss function (binary crossentropy with class weight balancing) and learning rate scheduler (step decay) were used during training for all MultiRes UNets for better comparability. For cycleGAN, the parameters used in the implementation of the authors in the original publication were used^23^. The threshold that has to be applied to the segmentation masks produced by the neural networks for binarization was varied between 0.0 and 1.0 in steps of 0.1, and the best results were used. Additionally, a watershed algorithm was applied to the binarized images prior to calculating the scores. For the segmentation masks produced by cycleGAN, the filtering process based on Li thresholding explained above for removing “artifact” particles was additionally applied after the watershed segmentation. For further details of image pre- and postprocessing see the Supporting Information.

The right choice of meaningful metrics for comparing segmentation masks for a specific task can be challenging and is still a subject of ongoing debates. The Jaccard index (or “intersection over union”, IoU) is often used for a first comparison, but for the task of particle segmentation, this metric might not be the most meaningful when “blindly” applied to the whole image. This is because fine details, e.g. fine boundaries between touching or overlapping particles in SEM images, often have very little influence on the overall Jaccard index but are very important for extracting meaningful particle size and shape descriptors from the segmentation results. For this reason, we also calculated an “instance-wise” Jaccard index where we calculated the IoU of each particle instance in the segmentation mask with each intersecting particle instance of the ground truth images, chose the best match, and averaged over all particle instances. Furthermore, we calculated receiver operating characteristic (ROC) curves for each of the six test cases and extracted an averaged Youden’s index. We also provide the results from metrics used in the ISBI challenge for the segmentation of neuronal structures in electron microscopy stacks^17^. Those include the pixel error (defined as 1—the maximal F-score of pixel similarity, or squared Euclidean distance between the original and the result labels), the minimum splits and mergers warping error (a segmentation metric that penalizes topological disagreements, in this case, the object splits and mergers^30^), the foreground-restricted Rand error (defined as 1—the maximal F-score of the foreground-restricted Rand index, a measure of similarity between two clusters or segmentations), as well as the later added foreground-restricted Rand scoring after border thinning and foreground-restricted information theoretic scoring after border thinning. While these metrics were originally used in a challenge for the segmentation of neuronal structures in electron microscopy images rather than nanoparticles, we believe that many of the same basic principles still apply. The results are summarized in Table 1. As can be seen, the UNet trained on simulated data outperforms the GAN in almost all measured categories.

**Figure 3:**
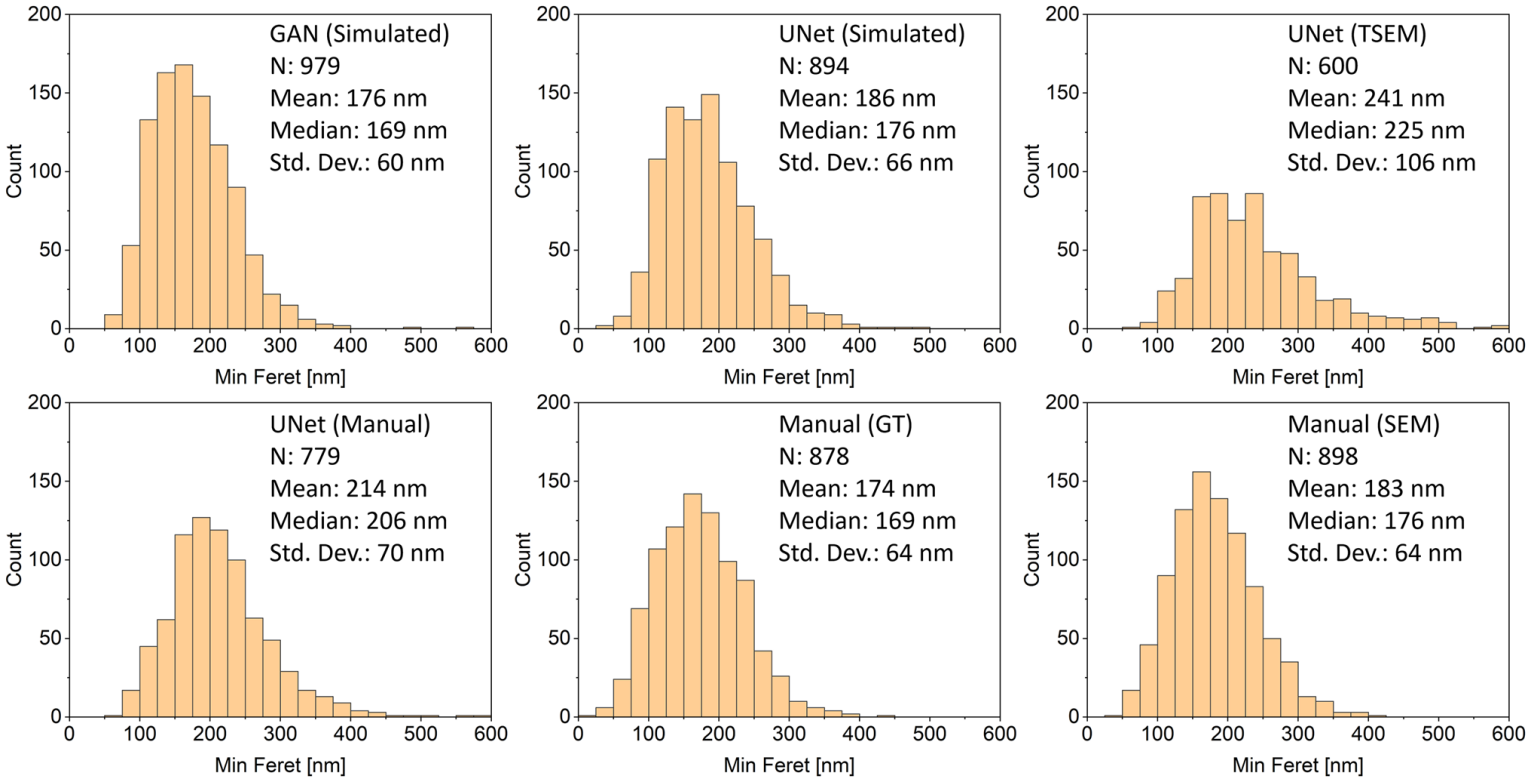
Size histograms (minimum Feret diameter) obtained with the methods mentioned in the text and in Table S1 (Supporting Information) for agglomerated titania nanoparticles.

Obtaining correct segmentation masks from the electron micrographs is certainly the most tedious and at the same time most challenging part of the image analysis, however, the masks are typically not the final endpoint or data of interest. In the case of SEM images of nanoparticles, the user is often interested in a quantitative description of the size, shape, or morphology of the imaged material. One of the most important and sought-after descriptors is the size and size distribution of the sample. Here the minimum Feret diameter is often of particular interest, given that—according to the Recommendation on the Definition of Nanomaterials by the European Commission—it is sufficient that one external dimension of a material in question is below 100 nm (number-based average) in order to be classified as a nanomaterial. It is noteworthy that measuring a size distribution from regular SEM images will always yield the sizes of 2D projections of the particles, with the size information in the direction along the z-axis missing. As long as the geometrical orientation of the particles is random and isotropic and enough particles are imaged, the minimum Feret diameter of the projections usually describes the smallest dimension reasonably well. In cases of highly anisotropic particle alignment on the substrate (such as flakes mostly parallel with the substrate) the smallest dimension might not be measured. For these cases more sophisticated imaging techniques such as electron tomography or correlative imaging by AFM would be necessary to accurately describe the real smallest dimension of the particle. Additionally it should be noted that the relatively straightforward calculation of the minimum Feret diameter (as well as many other size and shape descriptors such as maximum Feret diameter, perimeter, area, equivalent circular diameter, etc.) of every particle instance after segmentation reflects the correct particle measurements only in the case of single, non-agglomerated and non-overlapping particles that were correctly identified by the neural network, even when the individual particles are randomly oriented. In case a particle is partially occluded by another particle laying on top of it, the descriptors will give incorrect results even if the segmentation of the partially visible particle was done correctly. For this reason, most real-world applications will require an additional filtering step that removes partially occluded (and ideally also incorrectly identified) particles before further analyzing the segmentation masks. Different criteria can be used for this filtering process, such as the circumference, area, mean intensity, or solidity (i.e., the ratio between the particle area and the area of its convex hull) of the particle, however, for polydisperse samples or particles with complex shapes, finding suitable threshold values for these filters is often not trivial or might not be possible at all without introducing at least some bias that can skew the results. In some cases, the bias can be minimized by using an appropriate combination of several filters, but the choice of the most suitable filter combination and threshold values can be difficult and has often to be done manually. While this step is arguably far less time consuming and tedious than a manual segmentation of the image, it would still be desirable to automate this process as well. In the present case, we trained another MultiRes UNet for this classification task on manually classified images. This classifier network takes the original SEM image data and the segmentation mask generated by the segmentation network as input and gives the classification results as output. Each pixel in the output is classified as being part of a particle that should be kept for further analysis or discarded, then the average class score is calculated for each particle instance of the original segmentation mask, and particles with a score above a certain threshold (e.g. 0.5) are kept for further analysis. We trained such a classifier network on manually annotated particle classes and then used it to process the outputs of the four models shown in Table 1 to generate minimum Feret particle size distributions. For comparison, results obtained from the ground truth segmentation masks as well as results from manual size measurements performed directly on the SEM images by an electron microscopy specialist are given as well. The results are summarized in Fig. 3 and Table S1 (Supporting Information) for the minimum Feret diameter, as well as in Tables S2–S5 in the Supporting Information for other shape descriptors, namely maximum Feret diameter, perimeter, area, and equivalent circular diameter.

As can be seen from the quantitative comparison, all methods besides the UNet trained on masks generated from TSEM images give comparable results that are very close to the manually obtained values. A similar trend is also observed for the other descriptors, for which the values provided by the networks trained on simulated data mostly showed a deviation of only 5–10% from the ground truth values (see Tables S1–S5 in the Supporting Information). It is noteworthy that there is also a difference of about 9 nm (corresponding to 5%) between the mean minimum Feret diameters obtained from the ground truth segmentation masks and the measurements obtained directly from SEM images by a different operator. However, given that the pixel size in the SEM images used in this study was approximately 7.3 nm, this difference of 9 nm corresponds to a deviation of only 1.2 pixels on average.

Considering that the segmentation provided by the UNet better matches the perceived particle outlines than the segmentation provided by the GAN (see for example areas indicated by red arrows in Fig. 2), it is interesting how similar the results are for the final size distribution obtained from both segmentation models. Apparently, the segmentation errors were mainly from particles that were later filtered out by the classification network and hence not considered for the final particle size statistics. This could be because (partially) occluded particles were harder to segment correctly by the GAN, or the GAN made more mistakes when correctly identifying particles. However, in both cases the classifier network later removed the wrongly segmented or identified particles, so neither case influenced the final results of the particle size distributions significantly. Another noteworthy fact is that the network trained on simulated data gave final size distributions that were even closer to the values obtained from the ground truth segmentation masks than the network that was trained on these ground truth masks directly. One possible reason could be that the network trained on simulated data used a much larger training set than the network that was trained on the ground truth masks directly (1000 images vs. 34 images before tiling and augmentation), which might have enabled it to learn more segmentation details. On the other hand, the metrics shown in Table 1 show that the network that was trained directly on the ground truth masks performed better according to these metrics, which again suggests a better overall segmentation performance, but a worse performance for “relevant” particles. Similar observations can be made for the network that was trained on automatically generated masks from TSEM images, for which the particle size distribution deviates the most (qualitatively and quantitatively) from the ground truth despite a good performance when looking at the metrics provided in Table 1.

## Conclusion and outlook

In conclusion, we presented a workflow that can provide fully automated particle segmentation from scanning electron microscopy (SEM) images of agglomerated particles of complex shape, requiring minimal user interaction. A user only has to provide the SEM images and some exemplary particle shape outlines. Those are then used to generate more particle shapes from random noise by a first GAN, which are subsequently assembled into full segmentation masks. The latter are used together with the SEM images as input for unpaired image-to-image translations performed by a cycleGAN. The fully trained cycleGAN can then either be used directly for the segmentation of similar SEM images, or it can be used to generate a large amount of training data for training more sophisticated image segmentation networks (after an additional filtering step). While a qualitative and quantitative comparison of both models using a visual inspection of the segmentation masks as well as various metrics suggested better results when using a MultiRes UNet for segmentation, various size and shape descriptors obtained from the corresponding segmentation masks were very similar for both models, and in excellent agreement with manually obtained measurements. This can be explained by the fact that a second classifier network was used to filter out wrongly identified or overlapping or (partially) occluded particles that were unsuitable for obtaining meaningful measurements from the segmentation masks. These findings also highlight the importance of carefully choosing the evaluation metrics when comparing the performance of different methods or models, and the dataset provided here could serve as reference data for future benchmarking.

We anticipate that the same analysis process can be applied to a variety of different materials imaged by SEM without much fine-tuning. Furthermore, it might also be applicable for related segmentation tasks from images obtained by other methods such as TEM, light microscopy, AFM, or even CT or MRI. In fact, we did not optimize or tailor the various (hyper)parameters in the workflow to our specific problem and did not use any a priori knowledge concerning particle size and shape, other than providing “representative” particle outlines, i.e., outlines of differently shaped and sized particles. We used a standard implementation of the WGAN for obtaining random single particle masks, and for the cycleGAN we also used the standard parameters provided by the authors in their original publication. Parameters and methods such as the additional scaling factor, the number of particles per image, the thresholding algorithm, and the use of and parameters for the Perlin noise were also not the result of careful experimentation and fine-tuning, but rather chosen more or less arbitrarily after quick visual inspections of a few simulated segmentation masks.

Given the ability of GANs to also produce coherent image sequences (e.g. used for the generation of short video clips), the workflow might even be transferrable to 3D image segmentations. Finally, due to the modularity of the workflow, individual parts can be swapped out rather easily when better performing or more efficient algorithms or neural network architectures become available in the future.

## Methods

### Electron microscopy

SEM micrographs were recorded with an electron microscope of type Supra 40 from Zeiss (Oberkochen, Germany) equipped with a thermally assisted field emission source (Schottky filed emitter) and an InLens-SE-detector. Additionally, the transmission mode (STEM-in-SEM or simply TSEM) has been enabled by using a dedicated sample holder with the sample prepared as electron-transparent material on conventional TEM grids. The powder has been dissolved in 2 mL Millipore water, ultrasonicated for 15 min and 4 µL have been drop-casted on a conventional carbon TEM grid placed on filter paper and additionally blotted from the side with filer paper to get rid of liquid solvent.

TiO_2_ nanoparticles have been used in dry powder form as defined material of an inter-laboratory comparison for measuring nanomaterials in consumer products in the frame of the Horizon 2020 project ACEnano (www.acenano-project.eu/).

### Computational hardware and software

Neural Network training and inference was done on a Zotac GeForce RTX 2070 SUPER AMP! Extreme GPU with 8 GB of RAM in a HP Z8 G4 workstation PC equipped with a 1.80 GHz Intel Xeon Silver 4108 CPU and 32 GB of RAM. Python 3.7.4 and Keras with Tensorflow 2.2.0 were used throughout the project. For the ImageJ plugin to calculate various metrics, the FIJI distribution of ImageJ 1.52p was used. Data that were used for assessing and comparing neural network performance were part of the validation dataset and not used during training. See also the Supporting Information for more details.

The code used in this project, the fully trained neural networks, and the training and validation data including all SEM micrographs and their manually annotated segmentation masks are freely available as a GitHub repository. For added convenience, an ImageJ plugin that allows to use the fully trained neural networks in inference mode alongside some basic filters directly from ImageJ was implemented during this project as well, and the implementation is also made available on GitHub.

## Supplementary Information

Supplementary Information.
