# Supplementary material for: Workflow towards automated segmentation of agglomerated, non-spherical particles from electron microscopy images using artificial neural networks
Source: Sci Rep. 2021 Mar 2;11:4942. doi: 10.1038/s41598-021-84287-6 (PMC7925552; doi:10.1038/s41598-021-84287-6)
Supplement: Supplementary file 1 — Supplementary Information. [file 41598_2021_84287_MOESM1_ESM.doc]

Supporting Information

**Workflow towards Automated Segmentation of Agglomerated, Non-Spherical Particles from Electron Microscopy Images using Artificial Neural Networks**

Bastian Rühle*^,||^, Julian Frederic Krumrey^||,†^, Vasile-Dan Hodoroaba*^,§^

^||^Federal Institute for Materials Research and Testing (BAM), Richard-Willstätter-Strasse 11, D-12489 Berlin, Germany

^§^Federal Institute for Materials Research and Testing (BAM), Unter den Eichen 44-46, D-12203 Berlin, Germany

^†^ Present Address: Technical University of Berlin, Faculty IV - Electrical Engineering and Computer Science, Marchstrasse 23, 10587 Berlin

* [bastian.ruehle@bam.de](mailto:bastian.ruehle@bam.de), [dan.hodoroaba@bam.de](mailto:dan.hodoroaba@bam.de)

**Methods**

Available Files:

All the code used in this project, the fully trained neural networks, and the training and validation data including all SEM micrographs and their manually annotated segmentation masks, as well as an ImageJ plugin that allows to use the fully trained neural networks in inference mode alongside some basic filters directly from ImageJ are freely available on GitHub at <https://github.com/BAMresearch/automatic-sem-image-segmentation>

Technical notes on data pre- and post-processing:

The original SEM images were 1024 x 768 pixel, with the bottom 56 pixel rows containing metadata such as the calibration, acceleration voltage, working distance, etc. Hence, all images were cropped to a size of 1024 x 712 pixel before further use. These images were still too large for a standard 8 GB GPU however, so we decided to tile the images into 4 non-overlapping tiles rather than downscaling them to preserve their original resolution. Since the UNet model we used consisted of four blocks each in the encoder and decoder path that each reduce the image size by a factor of two, and since a vertical tile size of 356 pixel could not be reduced by a factor of 2^4^=16 without the need for additional padding, we used 512 x 352 pixel tiles instead of 512 x 356 pixel tiles, discarding the bottom 8 pixel rows in each image. The image tiles were further preprocessed by normalizing the values to the range of [0, 1], and augmented by including horizontally and/or vertically flipped versions of the tiles. Hence, each SEM image contributed 4x4=16 tiles to the training or validation dataset. The same preprocessing was used for the segmentation masks. All masks were also processed to ensure 4-connectivity rather than 8-connectivity, i.e., containing no diagonal segmentation lines. This was also necessary to reconcile differences in the way the particle detection algorithms of ImageJ and OpenCV operate. The WGAN implementation we used generated 64 x 64 pixel images of individual particle masks. In our case this size was sufficient for representative particle masks, especially considering the fact that they were later scaled again before being assembled into simulated segmentation masks, but in principle other sizes could be used here as well. For the cycleGAN, the same image tile size as for the UNet was chosen for convenience. Image tiles that contained very little or no particles at all (as determined by the mean pixel intensity of the tile not being at least 1.1 times the mean pixel intensity of the whole image) were not included in the training set of the cycleGAN. Also, we did not perform a training and validation split for the cycleGAN, but since it uses unpaired image-to-image translation, this should not result in an unfair bias or advantage, and even if it helped the GAN to better segment the images that were used for validation later on, this would be an advantage and not a hindrance in real-world applications. The images generated by the cycleGAN were not tiled further before using them for training the UNet because they already had the “correct” size of 512 x 352 pixel, but the same augmentations and normalization as before were applied.

When the models were used in inference mode, the images were tiled using overlapping tiles. On the one hand, this ensured that the whole image can be processed and the generated segmentation masks have the same size as the input, i.e., 1024 x 712 pixel and not just 1024 x 704 pixel, on the other hand it helped reducing stitching artifacts that can sometimes occur at the border of tiles. Nine 512 x 352 pixel tiles were spread out evenly across the images, run through the network, and the output was reassembled by taking the maximum in the overlapping regions. Afterwards, they were binarized using the Otsu threshold of the image and a watershed algorithm was applied. As revealed by the metrics that were calculated using variable thresholds, a threshold other than the one calculated by the Otsu method might have yielded slightly better results in individual cases, but since we were interested in automating the process as much as possible, we used the same thresholding for all models and all images. Also, the watershed algorithm sometimes introduced additional artifacts by adding segmentation lines the network did not predict, however, the overall benefits should still outweigh the drawbacks, because otherwise a single misclassified pixel could lead to the merging of two individual particle instances in the segmentation masks.

Details for automatically preparing masks from TSEM images:

For automatically binarizing the TSEM images to obtain segmentation masks for training the UNet, the TSEM images were processed by Otsu thresholding and watersheding. While the TSEM images were taken from the same sample position as the SEM images, in some cases there was a slight offset between both images, possibly due to sample drift or misalignment of the instrument. To better align the masks calculated from the TSEM images and the SEM images, we performed an image registration in ImageJ using the plugin “Register Virtual Stack Slices” after inverting the contrast of the TSEM images to help with feature extraction (TSEM shows dark particles on a bright background, while SEM shows bright particles on a dark background), using a translation model without deformations for both feature extraction and registration. The offsets are written to an xml file, from where they are parsed and applied to the masks and images during training of the UNet.

Details for manual measurements and annotations

Manual measurements of the minimum Feret diameters were performed by drawing a line across the smallest width of a particle as perceived by an electron microscopy specialist, and the length of the line was analyzed using the built-in tools available in ImageJ. The manual segmentation masks were prepared by outlining the perceived borders of each of the individual particles in each image using a custom-made python program. The classification of the fitness of the individual particle instances to be used in the statistics (i.e., excluding partially or mostly occluded instances which were outlined correctly, but are only partially visible and would hence give incorrect measurements) was performed using a custom-made ImageJ plugin that cycled through all instances and let the user assign a class to each instance individually. Next, the minimum Feret diameter and the other descriptors were determined for the “good” class automatically. Since all three processes were done manually and involved human judgement, they are not completely free of subjectivity, ambiguity, operator bias, and maybe even errors, but we discussed especially ambiguous cases and did our best to provide high quality annotated data. Indeed, the values for the minimum Feret diameters from two different manual methods performed by different persons agree very well with each other, showing an average deviation of 9 nm and 7 nm (or 1.2 pixels and 1.0 pixels) for the mean and median, respectively.

Details for time requirements of the individual steps:

Some typical time requirements for the individual steps on our computational setup (as described in the Methods part of the main manuscript) are given below. Please note however that the main goal of the work was not to minimize the overall processing time of the entire process (including training and inference), but rather to minimize user interaction time by automating as many steps of the process as possible, to reduce or eliminate operator bias, and to improve reproducibility and reliability of image analysis. By tuning the hyperparameters, especially the learning rates and schedules, the size of the training and validation sets, the batch size, and the number of training epochs, and by further optimizing the entire algorithm, the overall process could potentially be sped up considerably with only minimal losses in accuracy. Moreover, we would like to point out that the most time-consuming part, i.e. the training of the neural networks, only has to be done once and once a fully trained network is available, inference only takes a couple of seconds, meaning that any new images of the same or a similar sample can be processed considerably faster than by any manual process.

| Step | Typical Time Requirement |
| --- | --- |
| Preparing representative masks of individual particles (40 masks) | ~10-15 min^a)^ |
| Training the WGAN | 11 min |
| Generating simulated segmentation Masks (1000 images) | 34 min |
| Training the cycleGAN | 3 h 38 min |
| Generating and filtering corresponding SEM images (1000 images) | 4 min |
| Training the MultiRes UNet | 6 h 57 min |
| Generating segmentation masks with complete particle outlines that can be used for obtaining multiple descriptors (40 images) | 6 min |
|  |  |
| Manually measuring minimum Feret Diameters only (898 particles from 5 different images) | ~1.5 h^a)^ |
| Preparing manually annotated segmentation masks with complete particle outlines that can be used for obtaining multiple descriptors (40 images) | ~20-25 h^a)^ |

^a)^Manual step requiring user interaction

**Figures and Tables**

Table S1: Comparison of statistical parameters of particle size distributions (Minimum Feret diameter) for agglomerated titania nanoparticles.

| Method | Training Data | N | Mean (nm) | Median (nm) | Std. Dev. (nm) |
| --- | --- | --- | --- | --- | --- |
| GAN | Simulated | 979 | 176 | 169 | 60 |
| UNet | Simulated | 894 | 186 | 176 | 66 |
| UNet | TSEM | 600 | 241 | 225 | 106 |
| UNet | Ground Truth | 779 | 214 | 206 | 70 |
| Manual | Ground Truth^a)^ | 878 | 174 | 169 | 64 |
| Manual | SEM^b)^ | 898 | 183 | 176 | 64 |

^a)^ Measurements were performed directly on the same manually annotated masks that were also used as ground truth images when validating and testing the different neural networks. ^b)^ Measurements were performed directly on SEM images by manually drawing a line across individual particles and measuring its length.

Table S2: Comparison of statistical parameters of particle size distributions (Maximum Feret diameter) for agglomerated titania nanoparticles.

| Method | Training Data | N | Mean (nm) | Median (nm) | Std. Dev. (nm) |
| --- | --- | --- | --- | --- | --- |
| GAN | Simulated | 979 | 254 | 244 | 84 |
| UNet | Simulated | 894 | 265 | 257 | 91 |
| UNet | TSEM | 600 | 339 | 310 | 155 |
| UNet | Ground Truth | 779 | 300 | 288 | 102 |
| Manual | Ground Truth | 878 | 244 | 235 | 90 |

Table S3: Comparison of statistical parameters of particle perimeter for agglomerated titania nanoparticles.

| Method | Training Data | N | Mean (nm) | Median (nm) | Std. Dev. (nm) |
| --- | --- | --- | --- | --- | --- |
| GAN | Simulated | 979 | 760 | 715 | 284 |
| UNet | Simulated | 894 | 815 | 766 | 320 |
| UNet | TSEM | 600 | 1000 | 892 | 496 |
| UNet | Ground Truth | 779 | 880 | 835 | 310 |
| Manual | Ground Truth | 878 | 705 | 677 | 257 |

Table S4: Comparison of statistical parameters of particle area for agglomerated titania nanoparticles.

| Method | Training Data | N | Mean (nm^2^) | Median (nm^2^) | Std. Dev. (nm^2^) |
| --- | --- | --- | --- | --- | --- |
| GAN | Simulated | 979 | 33290 | 28233 | 25752 |
| UNet | Simulated | 894 | 36532 | 30228 | 27458 |
| UNet | TSEM | 600 | 66817 | 48160 | 99881 |
| UNet | Ground Truth | 779 | 49275 | 41392 | 34042 |
| Manual | Ground Truth | 878 | 34686 | 29338 | 25966 |

Table S5: Comparison of statistical parameters of particle size distributions (equivalent circular diameter) for agglomerated titania nanoparticles.

| Method | Training Data | N | Mean (nm) | Median (nm) | Std. Dev. (nm) |
| --- | --- | --- | --- | --- | --- |
| GAN | Simulated | 979 | 196 | 190 | 63 |
| UNet | Simulated | 894 | 205 | 196 | 68 |
| UNet | TSEM | 600 | 269 | 248 | 113 |
| UNet | Ground Truth | 779 | 239 | 230 | 76 |
| Manual | Ground Truth | 878 | 198 | 193 | 70 |


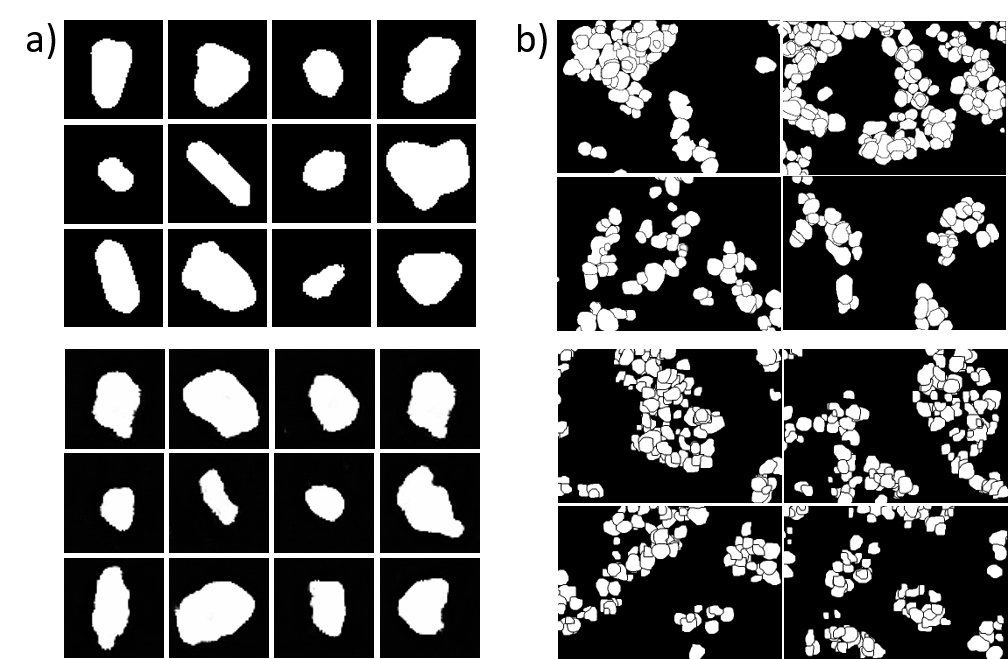


**Figure S2.** Generation of segmentation masks. a) Examples of real (top) and fake (bottom) masks of individual particles. The real masks have to be supplied by the user, the fake masks were generated by a Wasserstein GAN. b) Examples of real (top) and fake (bottom) segmentation masks. The fake masks were assembled randomly from single particle masks supplied by a Wasserstein GAN.
